# State of the art review of AI in renal imaging

**DOI:** 10.1007/s00261-025-04963-3

**Published:** 2025-04-28

**Authors:** Ali Sheikhy, Fatemeh Dehghani Firouzabadi, Nathan Lay, Negin Jarrah, Pouria Yazdian Anari, Ashkan Malayeri

**Affiliations:** 1 Radiology and Imaging Sciences, Clinical Center, National Institutes of Health, Bethesda, USA; 2 Department of Radiology and Biomedical Imaging, University of California, San Francisco, USA; 3 Artificial Intelligence Resource, National Institutes of Health, Bethesda, USA

**Keywords:** Renal mass, Artificial inteligence, Deep learning, Large language models, Computer assisted diagnosis

## Abstract

Renal cell carcinoma (RCC) as a significant health concern, with incidence rates rising annually due to increased use of cross-sectional imaging, leading to a higher detection of incidental renal lesions. Differentiation between benign and malignant renal lesions is essential for effective treatment planning and prognosis. Renal tumors present numerous histological subtypes with different prognoses, making precise subtype differentiation crucial. Artificial intelligence (AI), especially machine learning (ML) and deep learning (DL), shows promise in radiological analysis, providing advanced tools for renal lesion detection, segmentation, and classification to improve diagnosis and personalize treatment. Recent advancements in AI have demonstrated effectiveness in identifying renal lesions and predicting surveillance outcomes, yet limitations remain, including data variability, interpretability, and publication bias. In this review we explored the current role of AI in assessing kidney lesions, highlighting its potential in preoperative diagnosis and addressing existing challenges for clinical implementation.

## Introduction

In 2018, renal cell carcinoma (RCC) was responsible for around 175,000 cancer-related fatalities worldwide [1]. The incidence of RCC has steadily increased by about 2% annually over recent decades, mainly due to increasing use of cross-sectional imaging [2, 3]. According to some recent reports, incidental renal lesions are detected in about 27% of individuals who underwent an abdominal imaging study [4]. Accordingly, accurate and cost-effective differentiation of benign and malignant renal lesions is necessary. Renal tumors are classified into distinct histological subtypes based on their cellular origin and behavior, each with unique incidence rates, prognostic characteristics, and treatment options [5, 6]. Therefore, accurately distinguishing these subtypes using cross-sectional imaging remains challenging. Even after surgical removal, a significant proportion of renal tumors, particularly those smaller than 1 cm, are found to be benign upon histopathological examination [7, 8].

Recent developments in artificial intelligence (AI) have significantly enhanced radiological assessments, particularly through machine learning (ML) and its advanced deep learning (DL) techniques [9, 10]. Studies have demonstrated promising results for ML in detecting renal masses [11] and predicting surveillance outcomes beyond initial detection [12]. Additionally, recent advances, particularly the implementation of transformer-based models such as Vision Transformers (ViTs), have shown substantial improvements in diagnostic accuracy and image classification tasks within medical imaging. These transformer-based architectures leverage attention mechanisms, providing superior performance compared to traditional convolutional neural networks (CNNs), thus emerging as a promising direction for renal mass segmentation and subtype differentiation [13, 14].

**Fig. 1:**
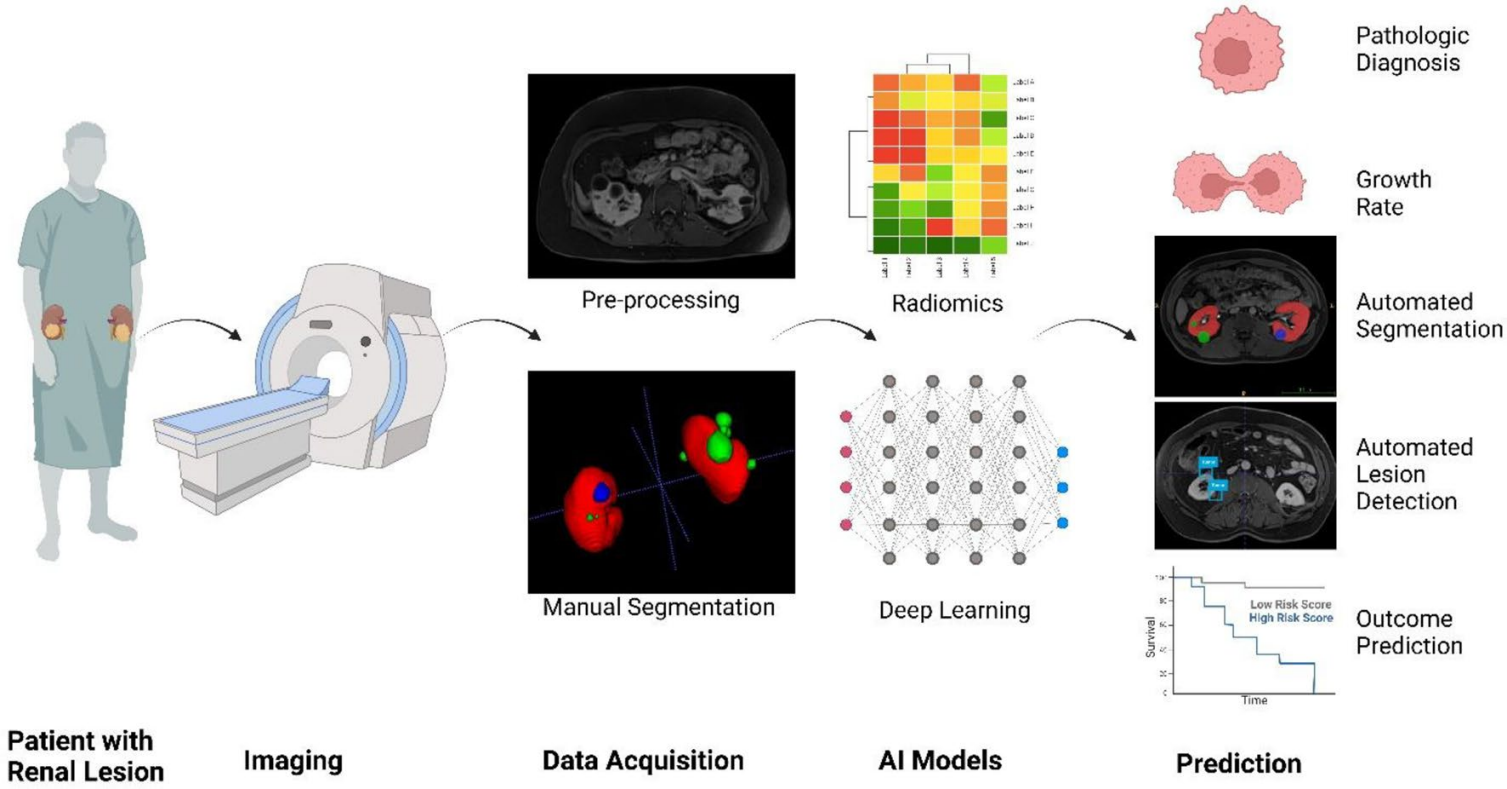
Overview of the workflow for AI-driven analysis of renal lesions [Created in BioRender. Sheikhy, A. (2024) https://BioRender.com/u29o302]

Computer-aided diagnosis (CAD) utilizing AI, particularly ML and its advanced deep learning (DL) technique, is emerging as a novel and promising field in radiological research. We begin with a review of the current role of AI in assessing kidney lesions and explore prospects in the preoperative diagnosis of renal lesions (Fig. 1).

## Renal tumors

The initial step in evaluating a renal mass involves distinguishing between benign cysts and solid masses. Most cystic renal masses are benign; even when malignant, they grow slowly. The Bosniak classification system is an established platform for categorizing cystic renal masses based on their risk of malignancy [15].

Up to 90% of solid renal tumors are malignant, including clear cell RCC (ccRCC), papillary RCC (papRCC), chromophobe RCC (chRCC), Collecting Duct Carcinoma (CDC), and Renal Medullary Carcinoma (RMC) despite their lower incidence compared to cystic tumors [16]. Angiomyolipoma (AML) and oncocytoma are the most common benign solid renal masses [17]. Imaging is crucial in diagnosing and characterizing renal masses, offering essential insights into staging, prognosis, therapeutic management, and follow-up [18]. Ultrasonography (US) can readily identify most incidental renal masses as simple cysts; however, it cannot consistently distinguish between benign and malignant solid renal tumors [19]. Most renal masses are discovered incidentally during baseline US or computed tomography (CT) scans in the venous phase, often performed for non-urological reasons [20]. Aside from the typical simple cyst and fat-containing AML, accurate characterization of these masses requires a specialized CT or MRI scan with intravenous contrast agents [21].

## Introduction and differences between AI, ML, and DL

Artificial Intelligence (AI) is a broad field of computer science that aims to create systems capable of performing tasks that typically require human intelligence. These tasks involve reasoning, problem-solving, interpreting natural language, and identifying patterns. In the past decade, AI and its subset, ML, have experienced a significant increase in their medical applications [22].

Machine learning (ML) is a field of computer science focused on allowing computers to “learn” without being explicitly programmed [23]. Machine learning can be broadly classified based on whether the computer’s learning is “supervised” or “unsupervised”. Supervised learning is analogous to the model-fitting techniques prevalent in epidemiologic research. In this approach, the outcome value, known as the “label” or “target” in machine learning, is specified for each observation. Such data with defined outcome values are termed “labeled data.” Supervised learning encompasses traditional epidemiologic methods, including linear and logistic regression, and widely used machine learning algorithms such as decision trees and support vector machines [24].

In unsupervised learning, the algorithm seeks to identify natural relationships and groupings within the data without reference to any specific outcome or “correct answer” [25].

The implication of ML methods is mainly on radiomics models, which aim to assess the growth rate [26], differentiate benign and malignant masses [27], and predict the risk of death [28].

Deep learning is a specialized area within machine learning that leverages multilayered artificial neural networks, commonly called “deep” networks. These networks enable deep learning algorithms to model and analyze intricate data patterns. This approach is particularly advantageous for tasks requiring complex manual feature extraction, such as image and speech recognition. Notably, the feature layers in deep learning are derived from the data rather than being manually manufactured by humans. Inspired by the human brain, this architecture has achieved notable success in visual recognition tasks, often surpassing human capabilities [29]. Artificial Neural Networks (ANNs) consist of interconnected artificial neurons organized in layers similar to the structure of the human brain. The backpropagation algorithm, crucial for training ANNs, adjusts the neurons’ weights to optimize performance. ANNs may exhibit some robustness to the dead neurons due to their distributed architecture [30].

Deep Learning (DL) plays a transformative role in renal imaging by enhancing the accuracy and efficiency of diagnosing and characterizing renal tumors. Using convolutional neural networks (CNNs) and other advanced neural architectures, DL algorithms can automatically segment [31], detect [11], and classify [32] renal lesions on medical images, including CT and MRI scans. These models excel in recognizing complex patterns and subtle differences in imaging data. DL methods like U-Net, YOLO, and Mask R-CNN are particularly effective in segmenting renal structures and differentiating between benign and malignant lesions, improving diagnostic accuracy and aiding personalized treatment planning. Additionally, DL algorithms facilitate non-invasive assessment of tumor characteristics, such as subtype classification and grading, potentially reducing the need for invasive procedures [32].

## Segmentation

Renal segmentation from MRI scans is a time-consuming phase of renal MRI studies [33]. In medical image analysis, image segmentation is a basic activity that is necessary to extract relevant objects or regions from an image to establish tissue characterization and improve diagnostic precision. In clinical practice, kidney segmentation is useful for assessing important parameters that can be utilized in clinical decision making, such as lesions size and volume. Additionally, it can be potentially helpful in evaluating best treatment strategies and planning for interventions. Manual region of interest (ROI) boundary tracing and stereology, conducted by a skilled expert with high experience, is the gold standard method of renal segmentation [34, 35]. The segmentation techniques are categorized as “manual segmentation”, “Image processing- and model-based segmentation”, and “ML/DL approaches”.

### Manual segmentation

Manual kidney segmentation accurately labels renal images during the training stage of a deep-learning workflow enterprise. It obtains the ground truth segmentation from which deep learning models are trained and benchmarked. However, this is an extremely time consuming process often requires an experienced radiologist to either prepare or inspect medical image segmentations [36]. Image editing applications, including ITK-SNAP, Analyze, Mango, MRIcron, 3D Slicer, Fiji, and Amira, are utilized in manual segmentation [37–43].

### Image processing and model-based segmentation

### Thresholding

**Fig. 2:**
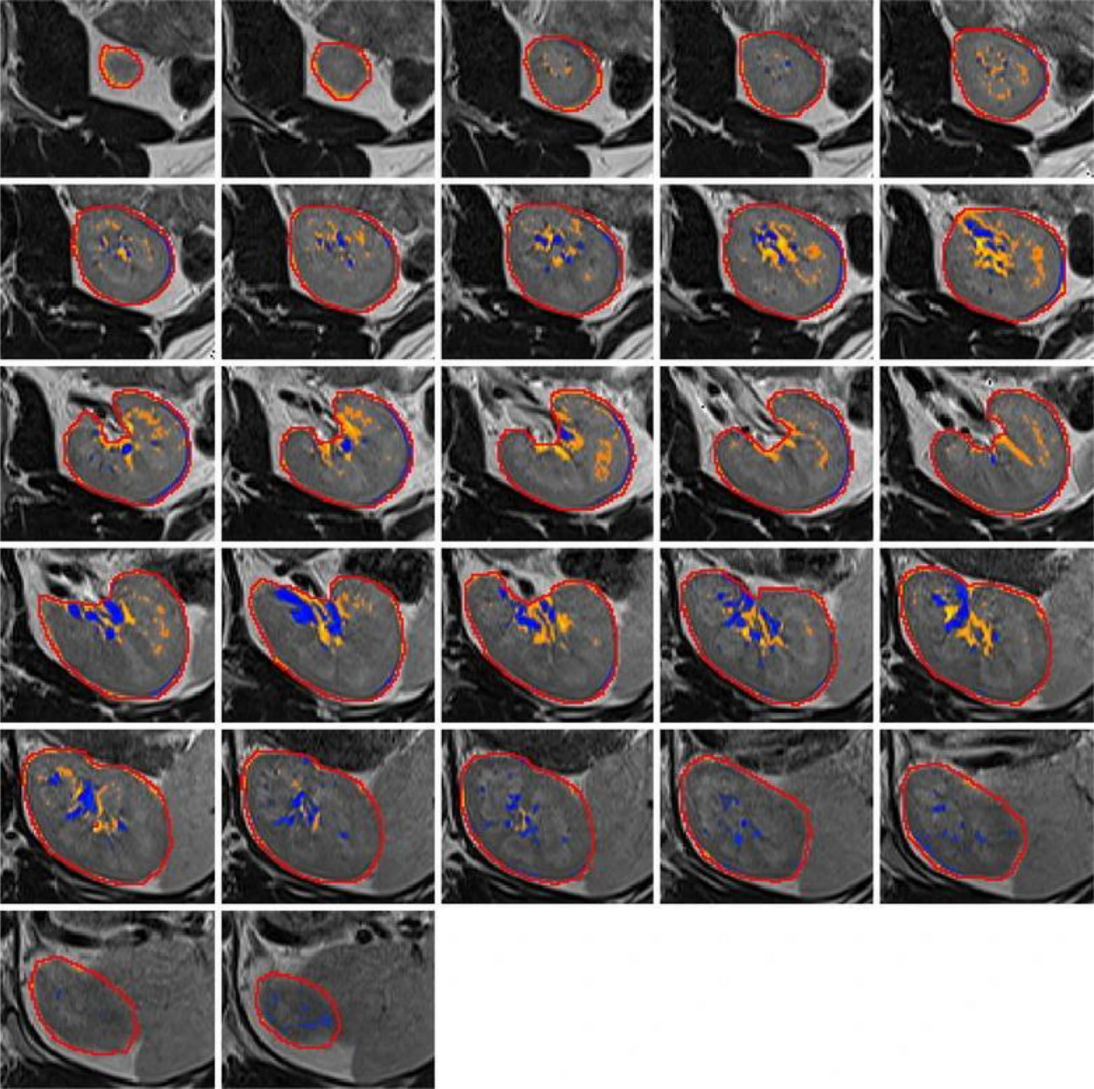
Study by Sandmair et al. [39] evaluated a semi-automatic unimodal thresholding method for volumetric analysis of the kidney in native T2-weighted MR images. In the kidney region of interest (axial slices), the red border indicates the manually defined pre-segmentation, while blue hues represent voxels below the lower threshold, and orange hues highlight voxels above the upper threshold

Unimodal thresholding was primarily proposed by Rosin et al. which is one of the simplest segmentation techniques, where pixel intensity values are compared to a threshold value to separate the regions of interest (e.g., kidneys, tumors) from the background [44]. Sandmair et al. utilized unimodal to assess total kidney volume (TKV) in patients with chronic kidney disease (CKD). Their results showed a 1.5 ml difference between the volumes acquired by semi-automated segmentation (Fig. 2) and the reference volume, which measured through manual segmentation as the gold standard [39].

### Region growing

Region growing starts from seed points selected within the kidney. The algorithm learns its homogeneity criterion automatically from the characteristics of the region. It expands by adding neighboring pixels with similar intensity or texture until the entire kidney or lesion is segmented [45]. Bae et al. conducted a study on individuals with renal cysts. According to their results, the region-based model had a relative bias of no more than 2.2% for cyst counts and less than 9.1% for volumes. The major limitation of this method is that manual intervention may still be necessary after the initial computer-based segmentation of individual renal cysts. A radiologist should manually outline their perimeters on the MR images if the program fails to segment certain cysts automatically. This process can include adding a new seed point to include a missed cyst or removing an existing seed point to eliminate an erroneously segmented structure [46].

### Edge detection

Edge detection is a fundamental technique utilized in many image processing applications to gather information from images as an initial step before feature extraction and object segmentation. This method identifies the contours of objects and the borders between objects and the background within an image [47]. An edge represents the boundary between two homogeneous areas within an image. Edge detection involves identifying and pinpointing the sharp transitions in an image. Gui et al. used renal mass edge detection as part of a tumor segmentation approach in ultrasound imaging [48]. Their results highlighted that they achieved the DICE coefficient of 90.16% and 91.13% for renal cysts and carcinomas, respectively.

### Watershed segmentation

Watershed segmentation was first proposed by Beucher et al. in 1979 as a segmentation method [49]. Watershed segmentation is an advanced image-processing methodology that exploits the analogy of geographic watershed dynamics to partition an image into distinct regions. In this technique, pixel intensities are interpreted as topographical elevations, where higher intensities correspond to peaks and lower intensities to valleys. The segmentation process initiates at the local minima within the image, which serves as the origins of simulated hydrological flooding [50]. As these regions expand, akin to water filling a basin, they encounter adjacent expanding regions, forming watershed lines at their interfaces. These lines establish the boundaries between different objects or areas within the image. Watershed segmentation is particularly effective in resolving complex, adjacent structures in fields such as medical imaging, where precision in spatial delineation is crucial. Despite its utility, the technique is susceptible to over-segmentation due to inherent noise and fine variations in intensity. It often requires additional refinement through merging strategies or the strategic placement of markers before segmentation to enhance the segmentation outcome [50].

Schmidt et al. utilized the 3D watershed segmentation method to separate multiple cysts that shared a boundary [51]. They also applied a global thresholding technique to refine the boundary.

### Active contour models (ACM)

The image segmentation algorithm based on Active Contour Models (ACM) is a sophisticated image processing technique that integrates high-level knowledge and prior inputs to achieve stable segmentation outcomes. This method enhances segmentation by incorporating grayscale and edge information during optimization. It delivers a piece-wise smooth, closed contour as the final result, characterized by its ability to adapt to diverse forms and flexible structures. ACM frames the image segmentation challenge as a minimization problem centered around an energy function. During the segmentation process, the contour of the target object is represented using the zero-level set, which facilitates handling topological changes during curve evolution. While the ability of ACM to automatically adapt the topology of segmented regions can be beneficial, it may also pose challenges depending on the specific application. Fundamentally, ACM uses a continuous, closed curve to delineate object boundaries, achieved through the standard gradient descent method to minimize the associated energy function [52]. Pandy et al. applied 3-D Unet and active contour for kidney tumor segmentation [53]. The first step of their method was the detection of the spine as a landmark for kidney region positioning. They applied an active contour segmentation method to delaminate the spine region in their images. Ultimately, they segmented kidneys using a convolutional neural network inspired by 3D U-Net.

### Level set (LS) method

The level set method is a numerical technique for tracking the evolution of interfaces. It serves an essential function in numerous image processing applications, such as image segmentation, reconstruction, and denoising [54]. Osher et al. first introduced the level-set method in 1988 [55].

Shehata et al. conducted a study on 3D kidney segmentation using diffusion-weighted magnetic resonance imaging (DW-MRI) [54]. According to their results, the DICE coefficients for the level set guided by image intensity only and the level set guided by the combined intensity and spatial features were 48.82 ± 6.32% and 58.19 ± 4.38%, respectively.

### Image registration-based segmentation

Atlas-Based Segmentation (ABS) is a widely utilized method in medical image analysis that employs a pre-labeled reference image, or atlas, to facilitate the segmentation of anatomical structures in new scans. The process begins with image registration, where the atlas is aligned to the target image by accounting for variations in anatomy, such as shape, size, and orientation. Following alignment, labels from the atlas are propagated to the corresponding regions in the target image, enabling automated segmentation. Multi-atlas approaches, which utilize multiple atlases to represent anatomical variability across different populations, enhance accuracy and robustness. This method is particularly valuable in clinical applications such as organ delineation in MRI or CT scans, improving efficiency while reducing the need for manual segmentation [56]. Zhao et al. utilized Multi-Atlas Registration (MAR) to segment kidneys on Micro-CT images [57]. Their model had the Dice coefficient of 0.92 and 0.98 in their studied datasets.

### Deep learning-based segmentation

Deep learning frameworks such as U-net and Convolutional Neural Networks (CNN) are frequently utilized for analyzing organ segmentation in clinical images [58]. Deep Learning Frameworks (DLF), Volumetric Segmentation Techniques (VST), and Hybrid Methods (HM) are some example models of kidney segmentation.

CNN, or Convolutional Neural Network, is a deep neural network well-suited for image analysis tasks. Recent developments in techniques based on convolutional neural networks for automated kidney segmentation have shown promising outcomes [59–62]. Most common models are U-Net and nn-Unet [63, 64], YOLO v8 [62], Mask R-CNN [60], MobileNetV2-UNet [65], DenseUNet [61], ResUNet [66], InceptionV4-Unet [67], and VGG Net [68].

## Differentiation between renal masses

### Machine learning and radiomics

Various imaging techniques are essential for diagnosing renal cell carcinoma (RCC). Ultrasound is an effective screening tool, reliably differentiating between solid and cystic renal lesions. However, definitive diagnosis requires utilizing more sophisticated imaging techniques such as computed tomography (CT) or magnetic resonance imaging (MRI) [69]. Despite the advances in imaging techniques, the interpretation of these images heavily relies on the radiologist’s subjective expertise and experience [70]. Radiomics offers a solution by incorporating parameters such as voxel, texture, and histogram analysis to extract more detailed information from conventional CT or MRI images than is perceptible to the human eye, hence, potentially improving the diagnostic accuracy of the human observer [71]. This process involves steps like image acquisition, volume of interest identification, segmentation (using computer-aided edge detection followed by manual correction), quantitative data extraction, and database construction [72]. Radiomics will become an integral part of the approach in oncology, aiding in cancer detection, diagnostic and prognostic assessment, and treatment response monitoring.

One significant challenge in RCC diagnosis is differentiating between benign and malignant lesions to avoid overtreatment. Radiomics-based machine learning models can also serve as non-invasive tools that enhance clinical workflow [10]. With this approach, renal masses identified on MR images can be processed through the machine learning model, which provides a probability of benignity versus malignancy. This probability can then be considered alongside the patient’s comorbidities to determine the optimal course of action, whether active surveillance, biopsy, or resection. RCC includes three major subtypes: clear cell RCC (ccRCC), papillary RCC (papRCC), and chromophobe RCC (chRCC), which vary in cellularity and vascularity [73]. Clear cell RCC is the most aggressive and lethal, constituting 75% of all RCCs and having a high metastatic potential. Papillary and chromophobe RCCs are less common, making up 10–15% and 5% of RCCs, respectively, and generally have better survival rates [74]. Subtyping RCC is clinically important, as it guides the use of molecular targeted therapies. Several studies have reported the application of radiomics in RCC subtyping. Kocak et al. aimed to validate their findings externally, enabling model replication and algorithm generalization using CT images and machine learning (ML) algorithms. The artificial neural network (ANN) classifier with adaptive boosting demonstrated the highest accuracy, achieving 84.6% in distinguishing clear cell RCC (ccRCC) from other tumor types. Conversely, the support vector machine (SVM) classifier achieved the best performance (69.2% accuracy) in differentiating ccRCC from papillary RCC (papRCC) and chromophobe RCC (chRCC) while showing poorer performance in distinguishing ccRCC or chRCC from other subtypes [75].

A systematic review and meta-analysis by Mühlbauer et al. found promising results for distinguishing angiomyolipoma from RCC and oncocytoma from RCC, with log odds ratios of 2.89 and 3.08, respectively. A pooled analysis of 30 studies showed a log odds ratio of 3.17 (p < 0.001) in differentiating benign from malignant lesions [76]. Ma et al. demonstrated that radiomics-based evaluation outperformed conventional CT in distinguishing fat-poor angiomyolipoma from clear cell RCC in a study of 84 pathologically proven renal masses [77]. Additionally, Goh et al. identified CT texture analysis as an independent predictor of tumor progression and response to targeted therapy in a study on imaging follow-up under systemic treatment on portal venous phase enhancement CT [78]. Li et al. evaluated the benefits of radiomics for differentiating chromophobe RCC from renal oncocytoma using multiphase CT scans of 61 patients, applying five ML algorithms. All models demonstrated high diagnostic accuracy, especially when combining data from corticomedullary and nephrographic phases [79]. Nassiri et al. developed a radiomic-based ML algorithm tested on 684 patients, which effectively distinguished renal masses with an area under the curve (AUC) of 0.84 [27]. Several studies have shown the potential of ML and DL algorithms in classifying RCC subtypes, Fuhrman grade, and prognosis [28, 80–82].

Numerous studies have leveraged radiomics and machine learning with CT and MR imaging to differentiate renal masses. For instance, Hoang et al. derived radiomics features from multiphasic post-contrast MR images and utilized a random forest model to distinguish between benign and malignant renal masses. They specifically compared papillary RCCs with clear cell RCCs, oncocytomas with clear cell RCCs, and oncocytomas with both papillary and clear cell RCCs, achieving accuracies of 77.9%, 79.3%, and 77.9%, respectively. However, this study’s focused comparisons of histologic entities do not reflect clinical practice, where a broader differential diagnosis is considered [83]. Other studies have similarly employed MRI-based radiomics analysis to differentiate RCC subtypes, assuming that benign entities have been excluded [84, 85]. In a study by Razik et al., involving a cohort of 54 solid renal masses in 42 patients, radiomics analysis without machine learning on fat-suppressed T1, T2-weighted, DWI at b500 and b1000 s/mm^2^, ADC maps, and post-contrast corticomedullary and nephrogenic phase T1-VIBE images identified features that distinguish benign from malignant entities, achieving AUCs greater than 0.80. This study’s small cohort, predominantly composed of clear cell RCCs, may have skewed the results. Additionally, machine-learning approaches sometimes identify distinguishing features through brute-force methods without adequate statistical correction due to numerous comparisons [86]. Said et al. conducted an MRI-based radiomics analysis to differentiate RCC from benign renal masses, achieving an AUC of 0.73, although the study was underpowered for benign lesions [87]. Finally, a large study by Xi et al., involving 1162 renal masses, employed a deep learning model, achieving an overall accuracy of 0.70 [88].

In a study conducted by Uhlig et al., which evaluated a large cohort of patient with renal masses using multiphase CT from various imaging centers, an XGB algorithm achieved an AUC of 0.84 in the internal validation dataset for distinguishing different renal tumor subtypes using combined arterial and venous phases of contrast enhancement. They found that identifying oncocytomas posed the greatest challenge for the XGB algorithm, consistently showing the lowest AUCs across contrast enhancement phases. This difficulty likely stems from similar radiological features between oncocytomas and ccRCC, such as a morphologically comparable central scar and central necrosis, respectively [89].

In a meta-analysis, Firouzabadi et al. identified six studies that used radiomics to differentiate oncocytoma from other renal tumors, analyzing 1064 lesions in 1049 patients (288 oncocytoma lesions and 776 RCC lesions). The study revealed pooled sensitivity and specificity of 0.818 and 0.808 for detecting different RCC subtypes (clear cell RCC, chromophobe RCC, and papillary RCC) from oncocytoma. Specifically, for distinguishing oncocytoma from chromophobe RCC, the pooled sensitivity and specificity were 0.83 and 0.92, respectively. This study concluded that CT radiomics has high accuracy in differentiating RCCs from oncocytomas, including distinguishing chromophobe RCCs from oncocytomas [90].

Another study by Firouzabadi et al. showed a pooled sensitivity of 0.779 for detecting RCCs vs. fp-AML and 0.817 for distinguishing ccRCC vs. fp-AML. The pooled specificity was 0.933 for detecting RCCs vs. fp-AML and 0.926 for distinguishing ccRCC vs. fp-AML. They suggest that radiomics features derived from CT have high sensitivity and specificity in differentiating RCCs from fp-AML, especially for ccRCC versus fp-AML [91].

Previous systematic reviews highlighted the methodological quality of 30 studies on AI application for renal mass characterization, emphasizing the importance of AI for clinical integration [92–94].

Li et al. evaluated a CT radiomics model to differentiate ccRCC from other tumor subtypes. They explored the potential of radiogenomics by integrating imaging features with the von Hippel-Lindau (VHL) mutation gene. Their model, built using the most relevant texture features from the corticomedullary phase, achieved a high AUC of 0.95 with an accuracy of 92.9%, with five out of eight features strongly associated with the VHL mutation gene [95]. Raman et al. investigated CT texture analysis features incorporated into a random forest (RF) model to differentiate common renal masses, including ccRCC and papRCC. Their model showed high accuracy in categorizing ccRCCs (91% sensitivity and 97% specificity) and papRCCs (100% sensitivity and 98% specificity), suggesting CT texture analysis with RF modeling as a promising method for renal mass characterization. These studies collectively highlight the potential of advanced imaging techniques and machine learning algorithms in improving the differentiation and characterization of renal cell carcinoma subtypes, addressing clinical needs for accurate diagnosis and treatment planning [96]. Leng et al. investigated the impact of denoising heterogeneity scores to distinguish angiomyolipoma (AML) from various subtypes of renal cell carcinoma (RCC). They found that the heterogeneity scores effectively discriminated clear cell RCC (ccRCC) and papillary RCC (papRCC), and reducing noise further improved the Area Under the Curve (AUC) of their models [97]. Yan et al. explored the diagnostic performance of texture analysis for discriminating AML with minimal fat, ccRCC, and papRCC using CT scans. Their analysis showed excellent classification results, particularly distinguishing between ccRCC and papRCC using nonlinear discriminant analysis, regardless of the scan phase. They observed a trend towards better lesion classification with corticomedullary and nephrographic phase images [98]. Hoang et al. assessed quantitative texture parameters from MRI to differentiate between common subtypes of RCC, specifically ccRCC and papRCC, in small renal masses (< 4 cm). Among the 45 extracted imaging features, textures successfully differentiated papRCC from ccRCC with an accuracy of 77.9%, sensitivity of 65.5%, and specificity of 88% [83]. Li et al. employed volumetric histogram analysis from apparent diffusion coefficient (ADC) maps to characterize small renal masses (SRMs). Their combination of mean ADC and histogram values achieved the highest AUC of 0.851, with a sensitivity of 80.0% and specificity of 86.1%, indicating the potential of volumetric analysis in distinguishing between certain types of kidney masses [99]. Paschall et al. investigated volumetric whole lesion apparent diffusion coefficient (WL ADC) parameters for identifying RCC, specifically type I papillary RCC (papRCC) from ccRCC and oncocytoma. Their study demonstrated that WL ADC could effectively differentiate between papRCC and ccRCC (p < 0.001), achieving an AUC of 95.2%, sensitivity of 84.5%, and specificity of 93.1% [100].

Recent studies have explored the integration of artificial intelligence (AI) and radiogenomics to correlate imaging features with molecular markers in clear cell renal cell carcinoma (ccRCC), particularly focusing on mutations in genes such as VHL, PBRM1, and BAP1.

One notable study investigated the associations between computed tomography (CT) imaging features and mutations in VHL, PBRM1, SETD2, KDM5C, and BAP1 genes. The researchers found that VHL mutations were significantly associated with well-defined tumor margins, nodular enhancement, and visible intertumoral vascularity. Conversely, mutations in KDM5C and BAP1 were linked to renal vein invasion. These findings suggest that specific imaging characteristics can serve as non-invasive biomarkers for underlying genetic mutations in ccRCC. [101].

Another study focused on predicting BAP1 mutation status using CT radiomics. By extracting texture features from CT images and employing a Random Forest classifier, the model achieved an accuracy of 83%, sensitivity of 72%, specificity of 87%, and an area under the curve (AUC) of 0.77. This demonstrates the potential of AI-driven radiomics in non-invasively determining BAP1 mutation status, which is crucial for personalized treatment strategies in ccRCC [102].

Furthermore, a study utilizing machine learning-based quantitative CT texture analysis aimed to predict PBRM1 mutation status in ccRCC patients. The Random Forest algorithm accurately classified 95% of cases concerning PBRM1 mutation status, with an AUC value of 0.987. The sensitivity, specificity, and precision for predicting tumors with the PBRM1 mutation were 94.6%, 95.4%, and 94.6%, respectively. These results underscore the efficacy of AI in correlating imaging features with specific genetic mutations [103].

Collectively, these studies highlight the promising role of AI in integrating radiogenomics into renal imaging. By correlating imaging features with molecular markers such as VHL, PBRM1, and BAP1 mutations, AI facilitates non-invasive prediction of genetic profiles, paving the way for personalized treatment approaches in ccRCC.

Despite analyzing large data sets, the need to manually define quantitative metrics is radiomics’ limitation. Machine learning (ML) and deep learning (DL) algorithms enhance automation by utilizing AI pattern recognition for image analysis [104]. In prostate cancer, AI has successfully detected malignancies on multi-parametric MRI (mpMRI) with high accuracy and predicted cancer grading with precision comparable to expert radiologists [105, 106]. One limiting step for preparing raw data for ML analysis involves segmenting medical images, followed by annotating regions of interest by a human observer [107]. Features are then extracted based on histogram distribution, skewness, and kurtosis [108]. DL algorithms can further facilitate this process by extracting information from annotated data and identifying additional predictive features [104]. The model is validated by dividing the database into training and validation sets and testing on a separate data set. For clinical implementation, the model needs external validation [107]. The advancements in machine learning (ML) algorithms are significant, yet approximately 30% of studies still utilize traditional algorithms for comparison. Consequently, direct comparisons between these approaches have yet to be published. Additional data is needed to accurately evaluate and generalize the most effective ML algorithms for radiomics research.

### Deep learning

Yazdian et al. demonstrated that YOLOv7 can effectively detect kidney cancers. They assessed 326 patients with 1034 tumors across ten benchmarks representing seven pathologies, including sporadic and hereditary tumors. In the 2D evaluation, their average results included a Positive Predictive Value (PPV) of 0.69 ± 0.05, sensitivity of 0.39 ± 0.02, and F1 score of 0.43 ± 0.03. For the 2.5D evaluation, the average outcomes were a PPV of 0.72 ± 0.06, sensitivity of 0.61 ± 0.06, and F1 score of 0.66 ± 0.04. Their top-performing model achieved a 2.5D PPV of 0.75, sensitivity of 0.69, and F1 score of 0.72 [11].

Xu et al. compared radiomics-based models, including random forest and deep learning (DL), to radiologists’ evaluations in a study of 217 patients with confirmed renal tumors on both T2-weighted imaging (T2WI) and diffusion-weighted imaging (DWI). The combination of DL and radiomics across both imaging sets achieved the highest diagnostic accuracy, with AUCs of 0.925 for T2WI and 0.826 for DWI, outperforming the radiologists’ assessments, which had AUCs of 0.724 and 0.667, respectively [104].

Lin et al. [109] compared a deep learning model against experienced radiologists for classifying benign vs malignant renal tumors on MRI. In a multi-institution dataset of 1162 lesions, the AI achieved ~ 70% test accuracy vs ~ 60% for radiologist experts (not statistically different, *p* = 0.053). Notably, the AI’s sensitivity for malignancy was significantly higher (92% vs 80% for experts, *p* = 0.017). This study demonstrated that an AI (ResNet-based) can perform on par with seasoned radiologists in renal lesion characterization, even slightly outperforming them in sensitivity.

In the multicenter study by Dai et al., the deep learning model’s performance was directly measured against radiologists. On the external test set, the AI achieved an AUC of 0.80 versus 0.84 for subspecialty radiologists (difference not significant, *p* = 0.61) [110]. Similarly, on a prospective set of new cases, the model’s AUC ~ 0.87 closely matched radiologist performance (~ 0.92, p = 0.70). Even for very small tumors (< 1 cm), the AI remained comparable to experts (AUC 0.74 vs 0.81, p = 0.78). These head-to-head results indicate that state-of-the-art AI can match experienced radiologists in differentiating benign and malignant renal masses on CT, including challenging small lesions.

Han et al. also aimed for reproducibility and generalizability in their models to differentiate ccRCC, papRCC, and chRCC using CT images and machine learning. Their deep learning neural network achieved an overall area under the curve (AUC) of 0.9 across all RCC subtypes (0.93 for ccRCC, 0.91 for papRCC, and 0.87 for chRCC), indicating promising classification performance with relatively lower accuracy for chRCC subtyping [111].

Tanaka et al. utilized the Inception-v3 convolutional neural network (CNN) architecture to differentiate small (≤ 4 cm) renal masses on multiphase contrast-enhanced CT. Their results showed that the highest accuracy, with an AUC of 0.846, was achieved with corticomedullary phase images. Notably, the deep learning model’s accuracy was relatively higher than that of the radiologist in their study (AUC = 0.648) [70].

### Fuhrman grading of the masses

On 1982 Fuhrman et al. introduced a histological grading system, which evaluates the size, shape, and nucleolar prominence of tumor cell nuclei. The Fuhrman grade ranges from 1 to 4, with higher grades correlating with more aggressive and poorly differentiated tumors [112].

A significant association exists between Fuhrman grade and patient prognosis [113, 114]. Among various therapeutic strategies, radical surgery remains the primary and most effective treatment for curing RCC patients. Radiofrequency ablation and active surveillance can be considered alternative options for small renal masses and low-risk small renal masses [115, 116]. Therefore, preoperative assessment of RCC tumor aggressiveness is crucial for optimal treatment planning and selecting appropriate follow-up regimens [117].

Several strategies propose the preoperative non-invasive prediction of ccRCC Fuhrman grade. MRI-derived ADC values are known indicators of tumor activity, with multiple studies assessing their utility in distinguishing low- and high-grade clear cell RCC [118, 119]. These studies have shown that MRI has acceptable predictive accuracy in the preoperative detection of high-grade RCC (AUC = 0.80) [120]. However, MRI is not as widely available as CT, and the range of reported ADC values for ccRCC varies significantly in the literature [121, 122]. Therefore, the robustness and repeatability of MRI need further validation.

CT-based semiquantitative and quantitative studies have attempted to classify low- and high-grade ccRCC [123, 124]. These studies have demonstrated that CT is a promising method for classifying low- and high-grade ccRCC. The radiomics approach, which converts medical images into quantitative, high-dimensional, and mineable features, enables the prediction of tumor status. However, the abundance of predictive modeling techniques requires a selection process to choose the most appropriate one for predicting tumor status.

Shu et al. compared the radiological features of different Fuhrman grade ccRCCs and extracted 1029 radiomics features from corticomedullary and nephrographic CT scans. They found that 11 and 24 features correlated with Fuhrman grades, respectively. This analysis confirmed that radiomics can preoperatively assess the Fuhrman grade of kidney lesions [125]. A retrospective study of 290 patients with histologically confirmed 298 RCCs evaluated the entropy and texture quantification levels within renal tumors using CT imaging. The study found a significant increase in entropy values in clear cell carcinoma and higher Fuhrman grades [126].

A study by Enming Cui et al. developed ML models using MRI and CT scans to differentiate between high-grade and low-grade ccRCC. The models were trained on data from 440 patients and validated on an external dataset of 20 patients. The best-performing model achieved an accuracy of 73% for MRI and 79% for CT in internal validation. For external validation, the MRI-based model achieved an accuracy of 74%, while the CT-based model achieved 69% accuracy [127].

Zahergivar et al. demonstrated their model’s high effectiveness in predicting Fuhrman renal tumor grades in patients with ccRCC based on the results of 100 iterations of their stacked ensemble model. For low-grade tumor detection, they reported a sensitivity of 0.79, a PPV of 0.95, and an F1 score of 0.86. For high-grade tumor detection, they found a sensitivity of 0.78, a PPV of 0.39, and an F1 score of 0.52. The model’s strength lies in its robustness, as shown by consistent performance across multiple iterations and a good Matthews Correlation Coefficient, indicating its reliability in predicting both low and high-grade tumors [128].

Recent studies investigating the role of ML have also analyzed texture in MRI imaging. Typically, T2 and DWI windows are used, and previous research on RCC masses has shown that entropy at spatial scaling factors (SSF) on DWI, corticomedullary phase, and nephrographic phase are the best parameters for assessing RCC grading. Accordingly, Stanzione et al. developed five algorithms incorporating various MRI features to predict tumor grading, achieving an accuracy greater than 90% [84, 129].

Chen et al. [78] recently reported using machine learning technology to predict patients’ ccRCC prognosis based on clinicopathological data. Their study screened various clinicopathological characteristics and developed a survival prediction model using a machine-learning algorithm incorporating tumor stage, grade, and size. This machine learning-based prognostic model performed exceptionally well in distinguishing patients with high survival risk and could serve as an independent prognostic factor for ccRCC patients. Functional enrichment analysis showed that the machine learning-based risk score was significantly associated with several biological processes, including the cell cycle, cell division, and DNA repair, which are known to be related to the occurrence and development of ccRCC [130, 131].

Yin et al. developed and tested an ML model using contrast-enhanced computed tomography (CECT) images to predict the Fuhrman grade of ccRCC. In a study involving 25 patients, the SVMRadial, RF, and Bayesian models demonstrated the best prognostic ability to predict the Fuhrman grade of ccRCC based on radiomics from CECT images [132]. Additionally, Lin et al. [81] developed a machine learning model combining three phases of CECT, demonstrating superior performance compared to single-phase radiomics models in predicting Fuhrman grades. Ding et al. [123] integrated texture features from corticomedullary- and nephrographic-phase CECT images, employing LASSO to select critical features and a logistic regression model to differentiate high- from low-grade ccRCCs at nephrectomy. Mostafa et al. [133] utilized three-phase CT scans to construct radiomic models, employing various image processing techniques and classification algorithms (SVM, random forest, logistic regression) to discern Fuhrman grades.

Shu et al. [125] extracted 1029 radiomic features from corticomedullary and nephrographic phases, employing LASSO regression to select features and logistic regression to classify high- and low-grade ccRCCs. Feng et al. [134] utilized three-phase CECT to extract first-order image features, highlighting entropy as an independent texture feature for distinguishing Fuhrman grades, albeit without a comprehensive assessment of different phase combinations.

In a recent study, Zhou et al. [135] innovatively developed multi-phase-combined radiomics models by extracting 3D classical radiomics features and computing Contrast Enhancement Variation (CEV) features from the entire 3D tumor mass. Their approach introduced several innovations: (1) Utilization of radiomics features from four-phase CECT to construct multi-phase-combined models for ccRCC Fuhrman grading, leveraging theoretical relationships with tumor hemodynamics and micro-vessel density due to ccRCC’s highly angiogenic nature. (2) Introduction of 3D CEV features to quantitatively represent variations in classical radiomics features across different phases, potentially reflecting the enhancement and heterogeneity of ccRCC tumor masses. (3) Comprehensive investigation of classical radiomics features, CEV features, and their combinations, revealing the pivotal roles of first order, CEV, and GLCM feature types in ccRCC Fuhrman grading.

Several studies have shown that deep learning models based on CT or MRI are effectively used for grading ccRCC in routine clinical settings, achieving accuracies ranging from 73.7 to 88% [32, 136–138]. However, in a study by Bai et al., the model was trained on contrast-enhanced ultrasound (CEUS) images and achieved an accuracy of 77.9% on the test set, demonstrating that CEUS performs comparably to CT or MRI. Since the human eye on medical images cannot distinguish nuclear grade, the algorithm’s assistance is valuable in predicting nuclear grade through a practical and non-invasive method [139].

## Foundation models, large language models (LLM), and large vision-language model (LVLM)

**Fig. 3:**
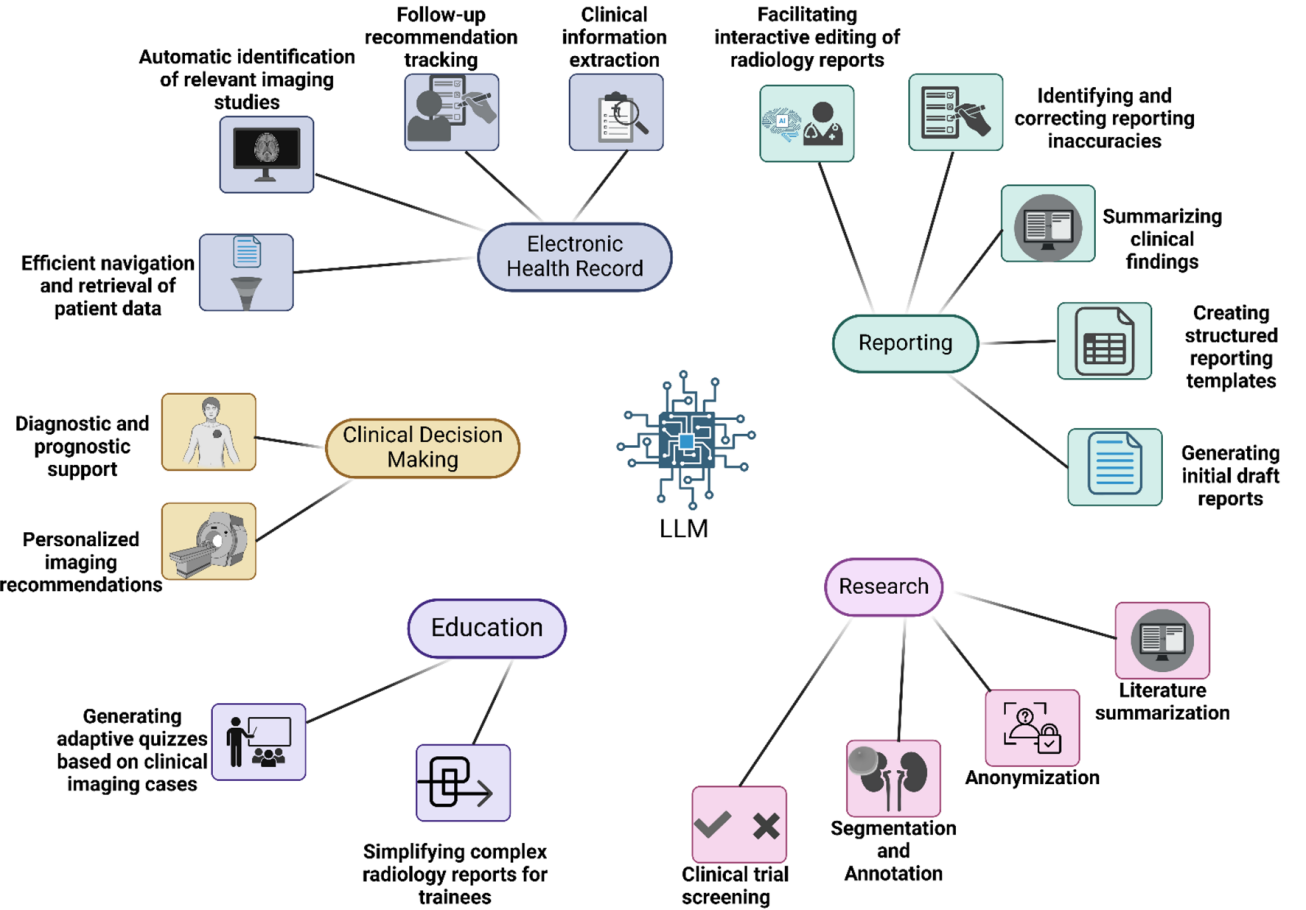
The application of Large Language Models (LLM) in radiology (Created in BioRender. Sheikhy, A. (2025) https://BioRender.com/j87k809)

Foundation models, large-scale AI models pre-trained on vast datasets, are rapidly advancing medical imaging (Fig. 3). Unlike traditional task-specific models, foundation models learn broad, transferable representations that can be adapted to various problems [140]. Recent breakthroughs in self-supervised learning enable these models to leverage unlabeled images, while transformer-based architectures and multimodal training (combining images with text or other data) equip them with “common sense” understanding of medical imaging [141].

### Applications in diagnosis

Pre-trained vision models can often identify abnormalities they were never explicitly trained to detect, exhibiting zero-shot diagnostic capabilities [142], for example, a recent vision-language foundation model called “Merlin” was trained on over 6 million abdominal CT images with paired radiology reports and clinical codes [143]. Without any task-specific re-training, Merlin can classify the presence of dozens of different imaging findings (e.g. hydronephrosis, renal masses) from CT scans in a zero-shot manner. In evaluations, this single model achieved an average F1 score of ~ 0.74 in recognizing 30 distinct abdominal findings, outperforming specialized radiologist-designed classifiers. Such results illustrate how large pre-trained models can generalize across diseases and even imaging modalities, detecting renal abnormalities with high sensitivity. Moreover, foundation models leveraging image-text joint training (inspired by models like CLIP) can associate medical images with textual descriptions [144].

LLMs and LVLMs are being leveraged to interpret renal imaging findings and assist in diagnosis. Vision-language models can analyze kidney images and identify pathologies such as tumors, cysts, or signs of disease. Notably, these models can identify subtle abnormalities that might be missed by radiologists [145]. For example, integration frameworks (e.g. ChatCAD) combine a large language model with specialized image analysis networks to interpret radiology images. the LLM ingests outputs from lesion detectors and classifiers and then produces a condensed diagnostic report in natural language [146]. This allows the model to provide decision support, summarizing findings and even offering recommendations. Early studies show that such LLM-driven systems can produce accurate, human-readable impressions of kidney imaging studies [147]. While current general-purpose models like GPT-4 with vision have limitations in direct clinical diagnosis [145], the trend indicates that LLMs will increasingly act as intelligent assistants to radiologists—cross-checking findings against vast medical knowledge and generating differential diagnoses or suggestions for further tests. This decision support role can help clinicians make more informed decisions, especially in complex renal cases.

### Application in segmentation

LVLMs also play a significant role in automating image segmentation for renal structures and lesions. Foundation models like Meta’s Segment Anything Model (SAM) [148] demonstrate prompt-driven segmentation, where the model can outline requested structures with minimal user input [149]. SAM has shown impressive zero-shot performance on multi-organ medical images, indicating that a single large model can generalize to segment kidneys or lesions without organ-specific training [150]. By integrating segmentation results with LLMs, systems can generate text descriptions of tumor size, location, and extent, which the LLM can incorporate into reports.

### Applications in prognosis and outcome prediction

By analyzing imaging patterns and combining them with clinical data, LLMs and LVLMs can help predict disease outcomes or progression. For example, researchers have shown that a multimodal large language model (taking both image and text inputs) could track and predict disease progression in a medical context [151]. In renal imaging, this could translate to predicting the likely growth of a kidney tumor or the decline in kidney function. Future LVLMs should evaluate imaging biomarkers (like lesion texture or perfusion patterns) to estimate tumor aggressiveness or patient survival probabilities. By providing risk stratification and prognostic insights, these models can assist in treatment planning (e.g. identifying which kidney tumors need urgent surgery versus active surveillance) and patient counseling.

### Applications in workflow automation

LLMs have the potential to streamline many steps of the renal imaging workflow, from image acquisition to report generation. One major application is automated report generation, which gets the imaging findings (either structured or free text), an LLM can draft a coherent radiology report or the impression section summarizing key conclusions [141]. Studies have found that GPT-4, for instance, can generate impressively realistic radiology report impressions from findings, closely matching human-written reports [152]. In practice, this could save radiologists time on documentation and ensure important findings (like a detected renal mass or hydronephrosis) are clearly noted. LLMs can also help extract information from prior reports or electronic health records—for example, pulling relevant patient history or lab results into a concise summary or automated extraction of actionable details of recommendations for additional imaging [153].

## Ensuring generalizability of AI models in renal imaging

AI models for renal imaging must generalize across different datasets, scanners, and clinical settings. This requires techniques to adapt models to new imaging protocols, train with multi-institution data, validate on diverse cohorts, and minimize scanner-related variability. Below we discuss key methodologies including, transfer learning, federated learning, multi-center validation, and image harmonization.

### Transfer learning for cross-protocol adaptation

Transfer learning leverages knowledge from one imaging dataset or protocol to improve performance on another. This is useful in renal imaging when a model trained on one scan protocol (e.g. a specific MRI sequence or CT phase) is adapted to a different protocol. A study pretrained a U-Net segmentation model on a contrast-enhanced T1-weighted MRI sequence and then fine-tuned it for five other MRI sequences (T2-weighted, in-phase, out-of-phase, pre-contrast, corticomedullary phase). This transfer learning approach boosted kidney segmentation accuracy, the average Dice score improved by about 3% compared to training separate models from scratch [154]. By reusing pretrained weights, the model could better handle the domain shift between imaging protocols, demonstrating improved generalizability across multiparametric MRI data.

### Federated learning for multi-institution training

Federated learning (FL) is an approach that enables multiple institutions to collaboratively train an AI model without sharing raw data, thereby preserving patient privacy. This technique has been identified as a way to produce more accurate, generalizable AI models by learning from a wide range of data sources [155]. For example, Kaissis et al. [156]demonstrated a privacy-preserving deep learning framework using federated learning on multi-institution medical imaging data.

### Multi-center validation of AI performance

Performing multi-center validation is crucial to verify that a renal imaging AI model generalizes beyond the data on which it was developed. Models are often initially trained on a single-institution or limited dataset, so testing them on external datasets from different hospitals or scanners provides a reality check on performance. A clear example is a multicenter study for automated kidney and liver volumetry in autosomal dominant polycystic kidney disease (ADPKD). Woznicki et al. trained a deep learning model on 992 MRI scans from multiple sites and then evaluated it on a separate test set of 93 patients as well as a large external cohort of 323 patients (831 MRI scans) from other centers [157]. The model showed high accuracy and robustness across this heterogeneous data as it was tested on scans from 40 different MRI scanners and maintained strong performance (DICE score 0.92–0.96). This high agreement across a large cohort indicates the AI’s reliability in diverse settings. These examples underscore that external validation on multi-center cohorts is a best practice—it helps identify any performance drop on new data and builds confidence that the AI will work consistently across different scanners, patient populations, and clinical workflows.

### Image harmonization to reduce scanner variability

Differences in scanner hardware and imaging protocols can make significant variability that challenges AI model generalizability. Image harmonization techniques aim to mitigate these scanner-related differences so that inputs from different sites appear more consistent to the model. One common statistical approach is the ComBat algorithm, originally developed to remove batch effects in high-dimensional data, which has been applied to medical imaging. For instance, in a multi-institution radiomics study of clear cell renal carcinoma, CT images were acquired from seven institutions using three different scanner vendors. The researchers applied ComBat harmonization to the extracted radiomic features, adjusting for site and scanner effects before modeling outcomes [158]. This step reduced the non-biological variability due to differing CT acquisition parameters, ensuring that the predictive model wasn’t confounded by which scanner produced a given image. Beyond statistical methods, deep learning offers powerful image-to-image translation tools for harmonization.

## Regulatory approval processes

FDA (United States): The FDA classifies AI-driven imaging tools as medical devices (often as Software as a Medical Device, SaMD) and subjects them to rigorous premarket review for safety and effectiveness. Between 2019 and mid-2023, the FDA authorized nearly 700 AI-enabled medical devices—over 75% in radiology. Many of these use pathways like 510(k) clearances or De Novo approvals, requiring evidence that the AI improves or is equivalent to standard care. The FDA also emphasizes Good Machine Learning Practice guidelines (developed jointly with UK’s MHRA and Health Canada) to ensure robust development and risk management for AI devices. Notably, the FDA has introduced the concept of “Predetermined Change Control Plans” (PCCP) to allow safe updates to learning algorithms post-approval. This is critical because AI models may evolve with new data, and regulators need a mechanism to oversee algorithm changes without requiring full re-approval each time**.**

CE Mark/EU (Europe): In Europe, AI imaging tools fall under the EU Medical Device Regulation (MDR 2017/745) and require a CE marking to be marketed. All but the lowest-risk software (Class I) must undergo assessment by a notified body. *Rule 11* of the MDR specifically ups the classification for most diagnostic software, meaning many AI diagnostic tools are at least Class IIa or higher. Compliance involves demonstrating clinical performance, risk controls, and fulfilling software lifecycle standards (like IEC 62304 for medical software). In practice, obtaining a CE mark is often seen as faster or less stringent than FDA approval [159]. Some companies launch AI products in Europe or other regions first, leveraging the CE mark, while continuing to pursue the more time-consuming FDA clearance [159].

## Current limitations and the future of AI

Despite its promise, radiomics faces several limitations that impact its clinical applicability and generalizability. First, the quality of input data and the parameters used in radiomics analysis can significantly impact the accuracy of results. For instance, signal intensity in MRI may vary even when scanning the same patient in identical positions, introducing variability that can affect the reliability of radiomic features [160]. Second, only a limited number of published radiomics studies are reproducible, largely due to the complexity and multi-step nature of the radiomics workflow. Inadequate reporting of study methodology and findings restricts both critical evaluation and the dissemination of results, underscoring the need for clear, detailed documentation, including supplementary data, code, and models [161]. Check List for Evaluation of Radiomics research or CLEAR guideline introduced by Kocak et al. on 2023 to address this issue [162]. Another limitation to the clinical application of radiomics models is the interpretability of the extracted features and models. Radiomics analyses are often described as a “black box,” meaning that the generated predictions are challenging to interpret in a clinically meaningful way [163]. Explainable AI (XAI) techniques, such as saliency maps, Grad-CAM, SHAP values, and attention mechanisms, can enhance transparency by highlighting key imaging features that drive AI predictions. However, these methods are not always reliable, and further research is needed to refine their interpretability and ensure consistency across different AI architectures. One of the major limitations of radiomics could be addressed as “publication bias”. Only a small fraction publishing negative findings. This publication bias is influenced by the tendency of editors and authors to prioritize and report positive outcomes, which are often perceived as more impactful and beneficial for career advancement. Hence, negative results, which is essential for ethical transparency and avoiding redundant research, frequently remain unpublished. These factors leads to a potentially inflated perception of radiomics’ effectiveness and reliability [164]. Moreover, overfitting remains a major concern, particularly in models trained on small or homogeneous datasets. Deep learning networks with excessive complexity can memorize training data rather than learning generalizable features, leading to poor performance on unseen cases. Regularization techniques, such as dropout layers, L2 normalization, and data augmentation, can mitigate overfitting, but external validation on independent datasets is crucial for confirming model robustness [165].

Dataset bias is another critical limitation, as AI models often learn patterns that reflect the characteristics of the training data rather than universal imaging features. For example, models trained on single-center datasets may underperform when applied to images acquired with different scanners, contrast agents, or patient populations. Federated learning and domain adaptation techniques can help address this issue by enabling multi-institutional model training without sharing sensitive patient data. However, achieving true generalizability requires greater diversity in training data, including underrepresented patient groups and rare renal tumor subtypes [166].

A critical factor in the success of AI algorithms is the quantity and diversity of training data. Deep learning models, particularly those used for renal imaging segmentation and classification, require large datasets with high-quality annotations to generalize well. In many cases, thousands of labeled images are needed to achieve robust performance [167]. However, in renal imaging, the availability of such datasets remains a challenge. Publicly available datasets are limited, making it necessary to rely on techniques such as transfer learning, data augmentation, or federated learning to improve model performance. Addressing this data scarcity issue is essential for translating AI research into clinical practice.

Prognostic analysis of tumor patients is a crucial application of deep learning research; however, current deep learning studies in RCC primarily focus on diagnosis and identification. There is a lack of research on predicting the prognosis of RCC patients. Additionally, studies examining the effectiveness of immunotherapy and targeted therapy for RCC patients remain limited.

Most studies conducted to date originate from a single medical center and lack validation in independent cohorts, resulting in biased outcomes and limited generalizability. Further multicenter, randomized controlled trials are needed to strengthen the robustness of findings. In addition, multidisciplinary and extensive collaboration is essential to actively advance deep learning research’s maturation, standardization, and clinical application.

AI models can assist in evaluating response to systemic therapies, such as targeted therapies or immunotherapy for metastatic renal cell carcinoma (RCC). By analyzing serial imaging scans, AI can detect subtle volumetric or texture-based changes in tumors, potentially identifying early signs of response or progression. Radiomics-based biomarkers, such as tumor heterogeneity, vascularity changes, and entropy measures, could complement conventional RECIST (Response Evaluation Criteria in Solid Tumors) criteria, providing more nuanced assessments of treatment efficacy [168].

AI can enhance longitudinal assessment by automating the detection and measurement of renal lesions over time. Deep learning-based segmentation models could track tumor growth or shrinkage across multiple imaging modalities (CT, MRI, ultrasound), reducing interobserver variability and improving early detection of tumor progression. AI-driven volumetric analysis may outperform traditional 2D diameter-based measurements, offering a more precise evaluation of lesion dynamics in both oncologic and nephrology settings [169].Radiogenomics, radiomics combined with genomics, uses preoperative images to identify specific genes in patients [170]. CT imaging can identify PBRM1 [171], BAP1 [172], and VHL [95] gene mutations in renal cell carcinomas. The potential to link imaging features with gene expression could significantly transform clinical practice and surgical strategies for renal lesions. However, achieving this goal requires further development, including larger and more comprehensive studies to enhance the applicability of radiogenomics and address current limitations associated with small cohort sizes and data heterogeneity.

Deep learning models have demonstrated remarkable success in the medical field, yet their lack of ability to explain poses a significant challenge. These models often comprise multiple layers of neural networks with numerous parameters and complex nonlinear mappings, resulting in opaque decision-making processes that obscure the rationale behind their predictions. This opacity can lead to concerns about trust and acceptance in clinical settings.

## Data Availability

No datasets were generated or analysed during the current study.
